# Fibulin-2 Is a Driver of Malignant Progression in Lung Adenocarcinoma

**DOI:** 10.1371/journal.pone.0067054

**Published:** 2013-06-10

**Authors:** Brandi N. Baird, Mark J. Schliekelman, Young-Ho Ahn, Yulong Chen, Jonathon D. Roybal, Bartley J. Gill, Dhruva K. Mishra, Baruch Erez, Michael O’Reilly, Yanan Yang, Mayuri Patel, Xin Liu, Nishan Thilaganathan, Irina V. Larina, Mary E. Dickinson, Jennifer L. West, Don L. Gibbons, Diane D. Liu, Min P. Kim, John M. Hicks, Ignacio I. Wistuba, Samir M. Hanash, Jonathan M. Kurie

**Affiliations:** 1 Department of Thoracic/Head and Neck Medical Oncology, University of Texas M D Anderson Cancer Center, Houston, Texas, United States of America; 2 Fred Hutchinson Cancer Research Center, Seattle, Washington, United States of America; 3 Department of Bioengineering, Rice University, Houston, Texas, United States of America,; 4 Department of Surgery, The Methodist Hospital Research Institute, Houston, Texas, United States of America; 5 Department of Radiation Oncology, University of Texas M D Anderson Cancer Center, Houston, Texas, United States of America; 6 Department of Molecular Physiology and Biophysics, Baylor College of Medicine, Houston, Texas, United States of America; 7 Department of Biostatistics, University of Texas M D Anderson Cancer Center, Houston, Texas, United States of America; 8 Texas Children’s Hospital, Houston, Texas, United States of America; Mayo Clinic College of Medicine, United States of America

## Abstract

The extracellular matrix of epithelial tumors undergoes structural remodeling during periods of uncontrolled growth, creating regional heterogeneity and torsional stress. How matrix integrity is maintained in the face of dynamic biophysical forces is largely undefined. Here we investigated the role of fibulin-2, a matrix glycoprotein that functions biomechanically as an inter-molecular clasp and thereby facilitates supra-molecular assembly. Fibulin-2 was abundant in the extracellular matrix of human lung adenocarcinomas and was highly expressed in tumor cell lines derived from mice that develop metastatic lung adenocarcinoma from co-expression of mutant K-ras and p53. Loss-of-function experiments in tumor cells revealed that fibulin-2 was required for tumor cells to grow and metastasize in syngeneic mice, a surprising finding given that other intra-tumoral cell types are known to secrete fibulin-2. However, tumor cells grew and metastasized equally well in *Fbln2*-null and -wild-type littermates, implying that malignant progression was dependent specifically upon tumor cell-derived fibulin-2, which could not be offset by other cellular sources of fibulin-2. Fibulin-2 deficiency impaired the ability of tumor cells to migrate and invade in Boyden chambers, to create a stiff extracellular matrix in mice, to cross-link secreted collagen, and to adhere to collagen. We conclude that fibulin-2 is a driver of malignant progression in lung adenocarcinoma and plays an unexpected role in collagen cross-linking and tumor cell adherence to collagen.

## Introduction

The extracellular matrix of epithelial tumors has features that differ sharply from those of adjacent tissue stroma [1]. Composed of a three-dimensional, collagen-rich scaffold that serves as a structural backbone for attached glycoproteins, sulfated proteoglycans, and loosely-tethered peptide ligands, tumor extracellular matrix evolves during malignant progression, developing collagen-dense fibrotic foci and enhanced stromal stiffness, which correlate with adverse prognosis and a higher risk of metastasis [2]. Tumor cells are important regulators of the tumor matrix; they secrete diverse intrinsic matrix molecules that integrate into the matrix scaffold and, in turn, serve as ligands for integrin receptors that drive tumor cell migration and invasion [3,4]. These findings support a bi-directional model in which tumor cells regulate the biochemical and biophysical properties of surrounding matrix, which governs tumor cell biological properties.

How tumor cells maintain extracellular matrix integrity in the face of dynamic matrix biochemical and biophysical forces is unclear. The fibulin gene family members (*FBLN1-5*) encode glycoproteins that play a central role in matrix stabilization by forming homodimeric complexes that fold into 3- or 4-armed structures, functioning as an inter-molecular clasp or buckle that stabilizes matrix macromolecules, including tropo-elastin fibers, microfibrils, and matrix-proteoglycan complexes [5–8]. Studies on mice deficient in fibulin family members have revealed a prominent role for fibulins in tissue repair. *Fbln2*-null mice have a normal lifespan under basal conditions and display no ultrastructural defects in extracellular matrix [9]. However, when stressed by myocardial infarction, *Fbln2*-null mice develop a phenotype of reduced cardiac tissue remodeling, attenuated TGF-β signaling within damaged cardiac tissues, and significantly improved survival [9], implying that fibulin-2 is required for repair of tissues damaged by hypoxic stress, a known driver of malignant progression of epithelial tumors [9]. Supporting a potential role for fibulin-2 in malignant progression, global transcriptomic profiling of diverse types of human adenocarcinoma revealed that fibulin-2 was more highly expressed in metastatic tumors than it was at primary sites [10], and a proteomic screen of highly and poorly metastatic tumor cell lines derived from mice that develop lung adenocarcinoma owing to co-expression of *Kras* ^G12D^ and *Trp53* ^R172HΔG^ (designated “KP” cells and mice, respectively) revealed that fibulin-2 was preferentially expressed in highly metastatic cells [11]. On the basis of these findings, here we posited that fibulin-2 drives malignant progression by stabilizing tumor extracellular matrix and tested this hypothesis by performing correlative studies on human lung adenocarcinomas and loss-of-function experiments on KP cells.

## Materials and Methods

### Immunohistochemistry

Mouse and human tissue specimens were fixed in 10% formalin and embedded in paraffin. Five µm paraffin sections were de-paraffinized with xylene and rehydrated with ethanol. Sections were then incubated in antigen retrieval solution (DAKO, Carpinteria, CA) for 20 minutes at 95°C followed by rinsing and blocking of endogenous peroxidase with 3% H_2_O_2_ in cold methanol for 20 minutes. Slides were rinsed and incubated in blocking buffer (5% BSA, 1% Skim Milk, 0.05% Triton X-100 in PBS) for 1 hour at room temperature. Sections were incubated overnight in a humidified chamber with antibodies against fibulin-2 (Novus Biologicals, Littleton, CO) at 1:250 dilution and then incubated with donkey anti-rabbit-HRP (Jackson ImmunoResearch, West Grove, PA) diluted 1:500 for 1 hour at room temperature. Slides were developed 3 to 5 minutes in liquid DAB substrate solution (DAKO) and counterstained with Meyer’s hematoxylin. Images were acquired using an Olympus IX71 inverted fluorescent microscope.

### Fibulin-2 expression in human lung adenocarcinomas

Immunohistochemical studies were carried out on tumor tissue specimens obtained from 46 surgically resected human lung adenocarcinomas, stages I-III, resected with curative intent. All cases were fully annotated for demographic variables, pathologic stage, smoking status and clinical outcome. Fibrillar and fibrous fibulin-2 staining was quantified separately in tumor, adjacent normal lung parenchyma, and lung tissue scars. The distribution of fibulin-2 expression was quantified in the peripheral areas (defined as the ~25% peripheral portion of the tumors) and central areas (defined as the ~75% central portion of the tumors).

### Murine lung adenocarcinoma cell lines

Murine lung adenocarcinoma cell lines (307P, 393P, 412P, 344SQ, 531LN2, 531LN3, and 344P) were isolated and established from primary or metastatic tumors of KP mice as described previously [12]. Cell lines were named according to the mouse number and site of derivation (e.g., 344SQ cells were from subcutaneous metastatic tumors of mouse 344). These cells have alveolar type II cell properties and variable propensities to undergo epithelial-to-mesenchymal transition and metastasize following injection into syngeneic mice [12]. Given that these murine cell lines are syngeneic, there are no reliable genotypic assays (e.g. DNA fingerprinting) for verification of cell line identity.

### Transfection of *Fbln2* short hairpin RNAs (shRNAs)

We sub-cloned murine *Fbln2*-specific shRNAs (Sigma-Aldrich, St. Louis, MO) and scrambled shRNA (Addgene, plasmid 1864) into lentiviral pLKO.1 TRC (Addgene, plasmid 10878). Additionally, pGIPZ lentiviral vectors containing *Fbln2*-specific shRNA sequences (V3LMM_515480 & V2LMM_26531) were purchased (Open Biosystems, Lafayette, CO). The target sequences for murine fibulin-2 were as follows: *Fbln2* shRNA #1, GGAGCAGAGGACAATGATA; #2, GCACTACCAGCTCAATTTC; #3, CCACTGTGTTCCTCAATTA; and #4, CGTCTCACTCTACAAGCAA. These vectors were packaged in 293T cells by co-transfection with packaging vectors psPAX2 (Addgene, plasmid 12260) and pMD2.G (Addgene, plasmid 12259). Cells (344SQ and 531LN2) were infected with viral particles and selected in puromycin (10 µg/mL) for up to 14 days to generate stable transfectants.

### Western blot analysis

Lysates from cell lines were harvested using RIPA buffer and were separated by SDS-PAGE and transferred onto a polyvinylidene fluoride nitrocellulose membrane (Bio-Rad Laboratories, Hercules, CA). Membranes were blocked overnight at 4°C in TBST with 5% nonfat dry milk followed by incubation with primary antibodies against fibulin-2 (Abcam, Cambridge, MA) and beta-actin as an internal control in TBST with 2.5% nonfat dry milk overnight at 4°C. Secondary antibodies linked to HRP were incubated for 1 hour at room temperature followed by detection with an enhanced chemiluminescence kit according to the manufacturer’s instructions (GE Healthcare, Pittsburgh, PA). Band intensities were quantified based on densitometry using ImageJ Software (http://rsbweb.nih.gov/ij/).

### Reverse transcription-polymerase chain reaction (RT-PCR) analysis

RNA was isolated from cells using Trizol Reagent (Invitrogen, Grand Island, NY), and 2 µg of each RNA sample was reverse-transcribed using qSCRIPT (Quanta Biosciences, Gaithersburg, MD). Quantitative RT-PCR analysis was performed using the ABI 7500 Fast Real-Time PCR System (Applied Biosystems, Foster City, CA) using the comparative threshold method with ribosomal protein *L32* mRNA as an endogenous reference housekeeping gene. For each reaction, a standard curve was performed using serial dilutions of a mixture of cDNA samples. SYBR green I (Applied Biosystems) was used as the fluorophore. Non-quantitative RT-PCR analysis of *Fbln2* transcripts was performed using Platinum Tag DNA polymerase (Invitrogen) with *L32* as a control. The primer sequences used are listed in Table S1.

### Cell proliferation in anchorage-dependent and -independent conditions

For cell proliferation assay, 1000 cells were seeded in 96-well plates, and then WST-1 assay was carried out at 4, 24, and 48 hours per manufacturer’s instructions (Millipore, Billerica, MA) with colorimetric readings taken at 450 nm. For anchorage independent cell growth, cells (5 x 10^4^ in 0.3% agar) were seeded onto a layer of 0.8% agar in 6-well plates, allowed to proliferate for 21 days, and stained with crystal violet. Colonies were visualized by light microscopy and scored per field of view.

### Migration and invasion assays

Cells in serum-free RPMI 1640 (5 x 10^4^) were seeded onto Transwell plates (for migration; BD Biosciences, San Jose, CA) or plates coated with growth-factor reduced Matrigel (for invasion) in the presence of mitomycin C (1 µg/mL), with RPMI 1640/10% FBS in the lower well as the chemo-attractant. After 16-18 hours of incubation, the medium was removed and the cells were fixed with 90% ethanol. The migrated cells were fixed, stained with 0.1% crystal violet, and then counted under the microscope.

### Cell adhesion assay

Cell adhesion on different ECM substrates was examined using 96-well plates pre-coated in triplicate with 5 µg/cm^2^ of corresponding ECM protein overnight at 4°C. Wells were washed with PBS and blocked with 0.5% BSA for 1 hour at room temperature. Cells were serum-starved for 1 hour prior to attachment assay at which point they were trypsinized and resuspended in serum free media at 5 x 10^4^ cells/mL. Cell suspensions (100 µL) were added to each well. Cells were allowed to adhere for 1 hour at 37°C followed by fixation and staining with 0.1% crystal violet, which was solubilized in 10% acetic acid, and quantified at 595 nm wavelength.

### Immunofluorescence

For chamber slide immunofluorescence, cells were cultured to confluence on 8-well glass Lab-Tek chamber slides (Thermo Scientific, Waltham, MA) and fixed in 1% formaldehyde for 30 minutes followed by blocking with serum-free protein block (DAKO) for 1 hour. Chamber slides were stained for one hour at room temperature with polyclonal rabbit antibodies against collagen type IV (Abcam) or laminin (Abcam) and rinsed in PBS (0.05% Tween 20). Sections were incubated with donkey anti-rabbit Alexa 594 (Invitrogen). Chamber wells were removed and slides were cover-slipped in Pro-Long Gold anti-fade mounting media containing DAPI (Invitrogen).

### Electron microscopy

Transmission electron microscopy was performed on glutaraldehyde-fixed, plastic embedded subcutaneous tumor tissue. The pseudopodial cytoplasmic projections along with other cellular features were assessed qualitatively at the ultrastructural level.

### Mechanical testing

For analysis of tissue compressive properties, lung disks of equal diameter were isolated by punch biopsy. Sample dimensions were measured using digital calipers prior to compression testing (n = 4 per formulation). Compressive testing was performed using an Instron Model 3340 mounted with a 10 N load cell. Instron Series IX/s software was used for testing control, and data acquisition as uniaxial compressive strain was applied at 0.2 mm/min. Force-elongation data was converted to stress-strain data with corrected cross-sectional area and plotted to derive the elastic modulus from the slope of the linear portion of the curve.

### Total and pepsin cleaved collagen content of cells in culture

Hydroxyproline measurements of total collagen were conducted according to manufacturer’s instructions (Cedar Lane Laboratories, Burlington, NC). Briefly, equal cell densities were plated and allowed to reach confluence for enhanced collagen deposition. Cell homogenates were isolated in equal volumes of H_2_O and diluted to a final concentration of 6 N HCl. Samples were hydrolyzed by baking at 110°C for 24 hours at which point floating lipid was removed and samples were evaporated using speed vacuum without heat and resuspend in equal volumes of H_2_O for subsequent oxidation with chloramine-T and quantification using DMAB substrate at 560 nm. Sircol measurements of solubilized collagen were performed according to manufacturer’s instructions (Biocolor, Carrickfergus, UK). Solubilized collagen fragments from pepsin-digested cell extracts (1 mg/mL gastric pepsin) were detected by Picrosirius red substrate and acid-salt wash followed by quantification at 555 nm.

### Animal husbandry and syngeneic tumor cell injections

As previously described [12], subcutaneous tumors were generated in syngeneic (129/SV), immunocompetent mice matched on the basis of age (2 to 4 months) and gender by injecting KP cells (1 x 10^6^) subcutaneously into the right flank in 100 µL PBS. Mice were monitored daily for 6 weeks, at which time necropsies were performed to isolate and weigh the primary tumors and count lung metastases. Intra-thoracic (orthotopic) injections were performed as previously described [13]. Briefly, 129/SV syngeneic mice (2 to 4 months) were injected in the left lung lobe with 20,000 cells suspended in 50 µL of 1:10 diluted growth-factor reduced Matrigel in Hanks’ balanced salt solution (BD Biosciences). Mice were monitored daily for 2 weeks, at which time necropsies were performed to isolate and weigh the lung tissue and assess lymph node metastasis.

### Ethics statement

Before their initiation, all mouse experiments were submitted to and approved by the Institutional Animal Care and Use Committee (IACUC) at the University of Texas M D Anderson Cancer Center (#02-09-01732). Mice received standards of care and were euthanized according to the standards set forth by the IACUC. A protocol to perform research on human tissues was submitted to the Office of Protocol Research and was reviewed, approved, and sanctioned by the Institutional Review Board of the University of Texas M D Anderson Cancer Center. Written informed consent was obtained from each patient.

### Statistical analysis

Statistical significance was determined by Student’s t-test. The difference was considered signiﬁcant at P < 0.05 (two-tailed).

## Results

### Distinct fibulin-2 expression patterns in human lung adenocarcinoma

**Figure 1:**
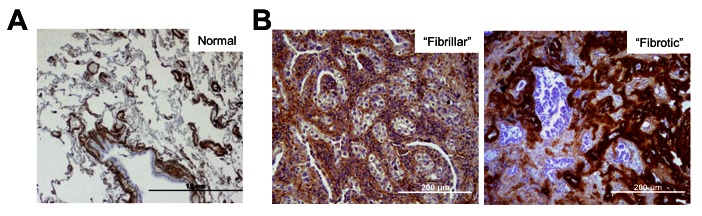
Distinct patterns of fibulin-2 expression in human lung adenocarcinomas and correlation with clinical outcome. Anti-fibulin-2 immunohistochemical staining (brown) of normal human lung adjacent to the tumor (A) and two different primary human lung adenocarcinomas representative of fibrillar (B, left panel) and fibrous (B, right panel) staining patterns. Size bars are indicated.

We examined whether fibulin-2 is expressed in human lung adenocarcinomas. Fibulin-2 was quantified by immunohistochemical analysis of 46 primary lung adenocarcinomas and adjacent lung tissues. Fibulin-2 was detected in elastic tissues within bronchi and vascular structures in adjacent normal lung (Figure 1A), in a “fibrillar” pattern between tumor cells and surrounding tumor acini, and in a “fibrotic” pattern within intra-tumoral fibrotic bands (Figure 1B). Most tumors exhibited both patterns; fibrillar staining was detected in all 46 tumors and fibrotic staining in 40.

### Fibulin-2 drives lung adenocarcinoma progression

**Figure 2:**
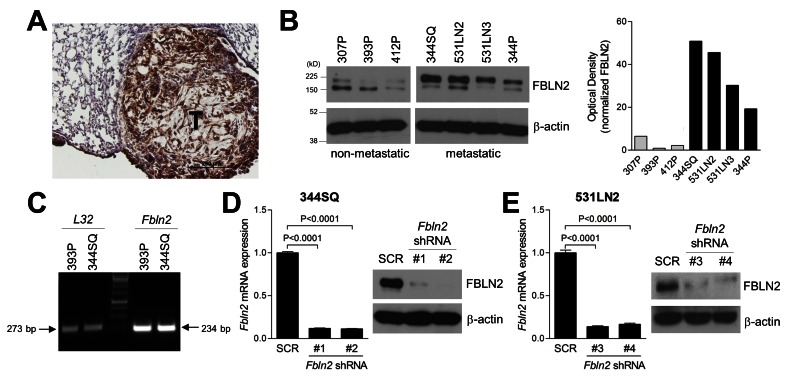
Fibulin-2 expression in murine lung adenocarcinomas and creation of fibulin-2-deficient cells. (A) A representative image of anti-fibulin-2 immunohistochemical staining (brown) in a spontaneous lung adenocarcinoma from a KP mouse. (B) Western blot analysis of fibulin-2 expression in metastatic (344SQ, 344P, 531LN2, and 531LN3) and non-metastatic (393P, 307P, and 412P) KP cell lines. Two fibulin-2 species were identified that differ on the basis of molecular weight (190 kD and 150 kD). β-actin included as loading control. Densitometric analysis of 190 kD band in western blot normalized on the basis of β-actin (bar graph). (C) Non-quantitative RT-PCR analysis of ribosomal protein *L32* (left) and *Fbln2* transcripts. (D and E) Quantitative RT-PCR (bar graph) and western blot (gels) assays on 344SQ cells (D) and 531LN2 cells (E) stably transfected with scrambled control shRNA (SCR) or one of four distinct *Fbln2* shRNAs (#1, #2, #3 or #4). RT-PCR values normalized on the basis of ribosomal protein *L32* mRNA and expressed as the mean values (± S.D.) of quadruplicate samples.

Fibulin-2 was detectable in spontaneous lung tumors in KP mice (Figure 2A) and in KP cells (Figure 2B). Western blotting of fibulin-2 in KP cells revealed two bands with molecular weights of approximately 190 kD and 150 kD; by densitometric analysis, the larger band was more abundant in metastatic KP cell lines (344SQ, 531LN2, 531LN3, and 344P) than it was in non-metastatic KP cell lines (307P, 393P, and 412P) (Figure 2B). *Fbln2* alternative splicing yields a transcript lacking exon 9 and generates a protein product 5 kD smaller than full-length fibulin-2 [5,6,8], whereas the two bands observed on fibulin-2 western blotting differed by approximately 40 kD (Figure 2B). Moreover, KP cells expressed a single *Fbln2* transcript that lacked exon 9 (Figure 2C), consistent with the presence of spliced but not full-length transcripts, arguing against differential alternative splicing in metastatic and non-metastatic KP cells.

**Figure 3:**
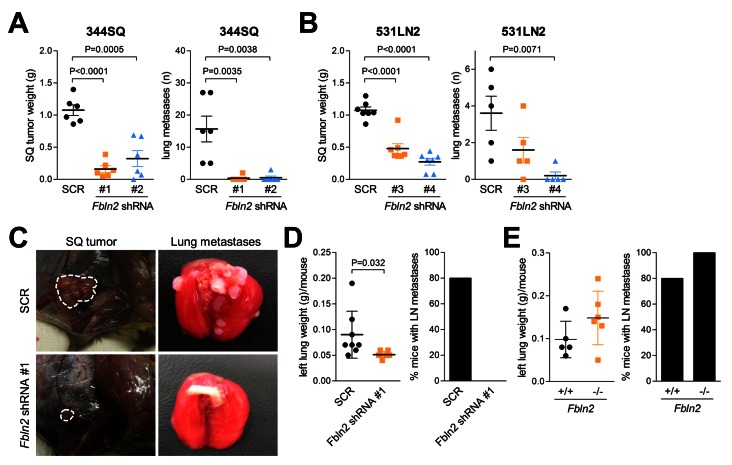
Fibulin-2 promotes the growth and metastatic properties of KP cells. (A–C) Transfectants (1 x 10^6^) injected into the flank of syngeneic wild-type mice (n=10 per cohort) to generate subcutaneous tumors. Mice sacrificed after 6 weeks to weigh primary tumor (left bar graphs in A and B) and count visible lung metastases (right bar graphs in A and B). Scatter plot representation of each tissue sample (squares), mean values (long line) and standard deviations (short line). P-values are indicated (t-test). Representative images of subcutaneous (SQ) tumors and lung metastases (C). (D) Syngeneic wild-type mice (n=10 per cohort) were injected intra-thoracically with transfectants (2 x 10^4^) to generate a single orthotopic lung tumor. Mice were sacrificed after 2 weeks to weigh left lung (bearing tumor) and determine the presence or absence of metastases to mediastinal lymph nodes. Scatter plot representation of each tissue sample (squares), mean values (long line) and standard deviations (short line). P-values are indicated (t-test). Percentages of mice in each cohort with metastases to mediastinal lymph nodes (bar graph). (E) *Fbln2*-null (-/-) and -wild-type (+/+) mice were injected intra-thoracically with 344SQ cells (2 x 10^4^) to generate a single orthotopic lung tumor and analyzed as described in (D). P-values are indicated (t-test).

To examine the role of fibulin-2 in lung tumorigenesis, two different metastatic KP cells (344SQ and 531LN2) were stably transfected with *Fbln2*-specific and scrambled shRNAs to generate fibulin-2-deficient and control transfectants, respectively (Figure 2D and E, and Figure S1). The transfectants were injected into syngeneic wild-type mice subcutaneously to generate flank tumors that metastasize predominately to the lungs (Figure 3A and B), or intra-thoracically to create orthotopic lung tumors that metastasize predominately to mediastinal nodes (Figure 3C and D) [12,13]. Relative to controls, the fibulin-2-deficient tumors were significantly smaller and produced fewer metastases regardless of injection site (Figure 3A–D).

Other intra-tumoral cell types such as fibroblasts, normal epithelial cells, and endothelial cells are known to express fibulin-2 [14,15]. To determine whether other cellular sources of fibulin-2 play a biological role during malignant progression, 344SQ cells were injected into *Fbln2*-null and -wild-type littermates [16]. Orthotopic lung tumors grew and metastasized in the setting of both genotypes (Figure 3E). Thus, malignant progression was dependent specifically upon tumor cell-derived fibulin-2, which could not be offset by other cellular sources of fibulin-2.

**Figure 4:**
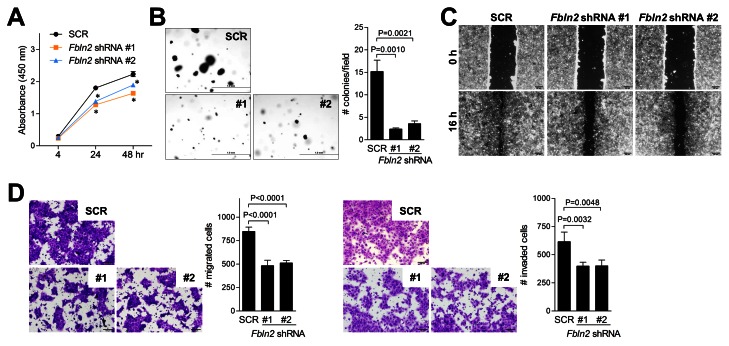
Fibulin-2 promotes 344SQ cell proliferation, migration, and invasion. (A) Cell proliferation (WST-1) assays to quantify relative cell density of transfectants grown in monolayer cultures. Results expressed as mean values of triplicate wells (± S.D.) at each time point. Asterisks indicate P < 0.01, SCR shRNA versus *Fbln2* shRNA. (B) Colonies of transfectants grown in soft agar were photographed and counted after 21 days. Results expressed as mean values (± S.D.) of triplicate wells (bar graph). (C) Images of confluent cultures subjected to scratch-wounds after 16 hours. (D) Migration and invasion assays of transfectants loaded into upper chambers of Boyden apparatus in the presence of 10% serum in bottom chamber as chemo-attractant and 1 µg/mL mitomycin C to block cell proliferation. Cells migrating or invading through the filters were stained with crystal violet, photographed, and quantified. Results represent the mean values (± S.D.) of quadruplicate samples (bar graph). P-values are indicated (t-test).

**Figure 5:**
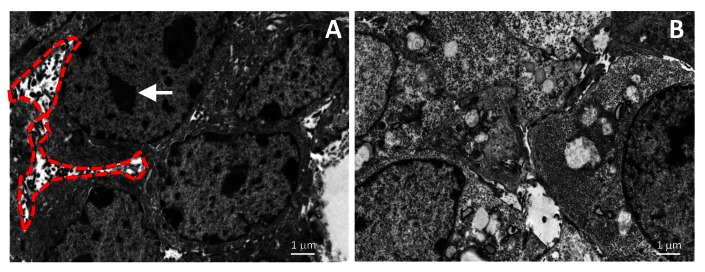
Fibulin-2 deficiency reduces tumor cell pseudopodia. Transmission electron microscopic images of subcutaneous tumors transfected with scrambled shRNA (A) or *Fbln2* shRNA (B). Pseudopodia (enclosed by dotted red line) and heterochromatin (arrow) are indicated.

On the basis of the above findings, we posited that tumor cell-derived fibulin-2 promotes the migratory, invasive, and proliferative capacity of tumor cells and performed *in vitro* studies on fibulin-2-deficient cells to test this hypothesis. Compared to controls, fibulin-2-deficient cells were less proliferative in monolayer cultures (Figure 4A), formed fewer colonies in soft agar (Figure 4B), displayed reduced migration in wound-healing assays (Figure 4C), and exhibited diminished migration and invasion in Boyden chambers (Figure 4D). Transmission electron microscopic analysis of control tumors revealed abundant pseudopodia and heterochromatin (Figure 5A), which were less abundant in fibulin-2-deficient tumors (Figure 5B), reflecting a loss of invasive and proliferative properties, respectively. Thus, fibulin-2 enhanced tumor cell proliferation, migration, and invasion, which could have mediated its promotion of tumorigenesis and metastasis.

### Fibulin-2 promotes cross-linking of secreted collagen and tumor cell adherence to collagen

Fibulin-2 is a nidogen-interacting protein and binds to the nidogen-like domain of MUC4, an interaction that has been implicated as a mediator of MUC4-induced invasion [17]. Given that MUC4 expression was detectable in KP cells (Figure S2), we examined whether fibulin-2 forms a complex with MUC4. However, MUC4 did not co-immunoprecipitate with fibulin-2 (Figure S2). Although physical interactions may occur at levels not detectable by this assay, these findings argue against a role for MUC4/fibulin-2 complexes in this model.

**Figure 6:**
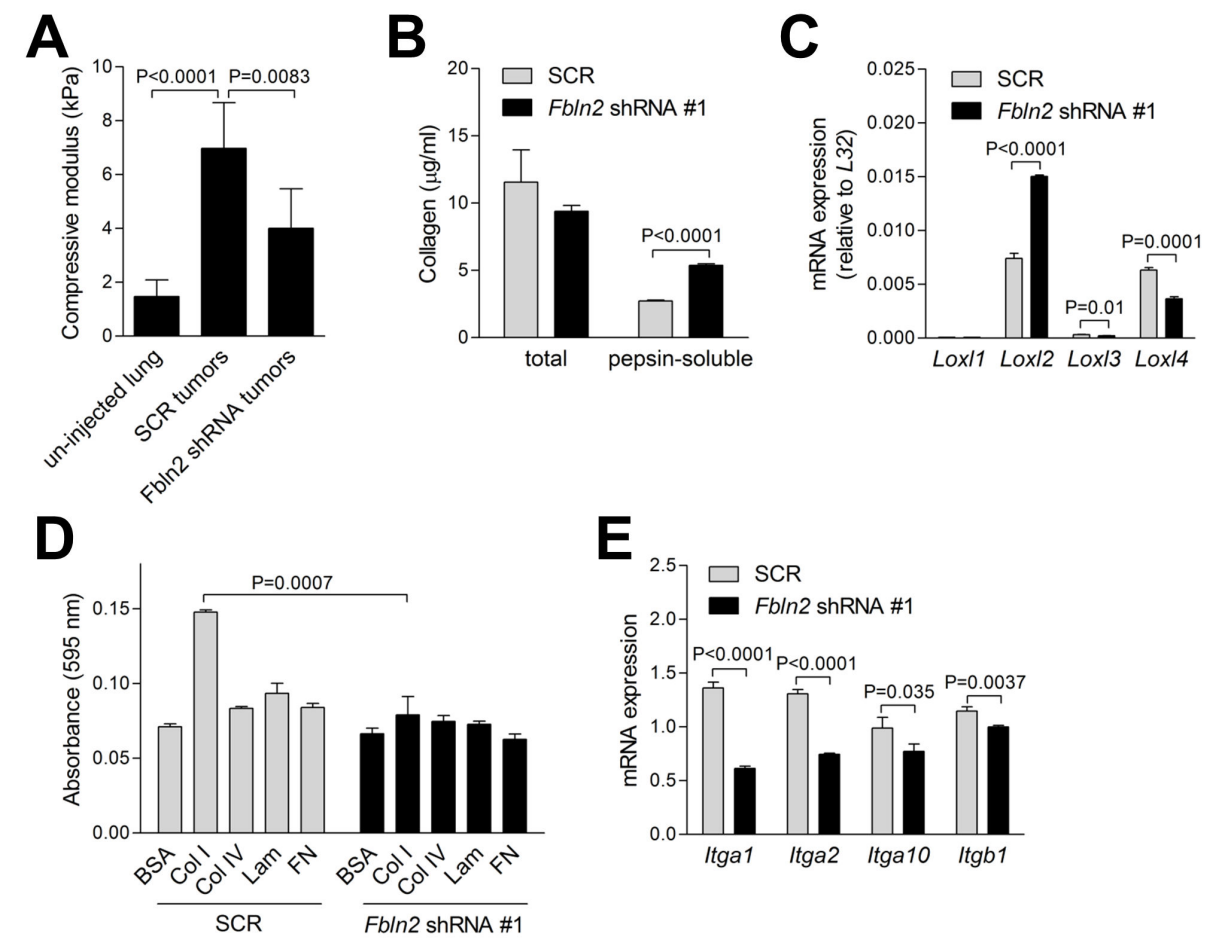
Fibulin-2 promotes cross-linking of secreted collagen and tumor cell adherence to collagen. (A) Compressive testing performed on punch biopsies of un-injected lungs (n=13) and 344SQ orthotopic lung tumors (*Fbln2* shRNA [n=9] or SCR shRNA [n=5] transfectants). Results represent mean values ± S.D. of replicate samples. (B) Quantification of pepsin-soluble and total collagen in conditioned media samples by sircol assays and hydroxyproline assays, respectively. Results represent mean values ± S.D. of triplicate samples. (C) Quantitative RT-PCR assays on 344SQ cells stably transfected with scrambled control shRNA (SCR) or *Fbln2* shRNA #1. Values normalized on the basis of ribosomal protein *L32* mRNA and expressed as the mean values (± S.D.) of quadruplicate samples. P-values are indicated (t-test). (D) Adherent cells were stained with crystal violet and quantified colorimetrically 1 h after seeding on plastic or coated with indicated matrix molecules or bovine serum albumin (BSA) as a control. Results expressed as mean values (± S.D.) of triplicate wells. (E) Quantitative RT-PCR assays on 344SQ cells stably transfected with scrambled control shRNA (SCR) or *Fbln2* shRNA #1. Values normalized on the basis of ribosomal protein *L32* and expressed as the mean values (± S.D.) of quadruplicate samples. P-values are indicated (t-test).

Invasion into adjacent stroma requires that tumor cells create mature extracellular matrix fibers and adhere to and migrate along those fibers [2]. We examined whether fibulin-2 deficiency abrogated the ability of tumor cells to perform these functions. Compression testing was performed on fibulin-2-deficient and -replete orthotopic lung tumors to quantify tissue stiffness, an indirect measure of cross-linked collagen, which revealed that stiffness was reduced in fibulin-2-deficient tumors (Figure 6A). Quantification of collagen concentrations in conditioned media samples from fibulin-2-deficient and -replete cells revealed no difference in total collagen, but soluble collagen was significantly more abundant in fibulin-2-deficient cells (Figure 6B), reflecting a reduction in cross-linked collagen. Analysis of enzymes that control collagen cross-linking revealed that *Loxl2* and *Loxl4* were the most abundantly expressed, and fibulin-2 deficiency reduced the expression of *Loxl4* but paradoxically increased *Loxl2* (Figure 6C). We examined tumor cell adherence to matrix molecules by seeding 344SQ cells in wells pre-coated with collagen type I, collagen type IV, laminin, or fibronectin. Cells adhered to collagen type I but not the other matrix molecules, and adherence to collagen type I was abrogated by fibulin-2 depletion (Figure 6D). Quantification of integrins that bind collagen (α1β1, α2β1, α10β1, and α11β1) [18] revealed significant reductions in *Itga1, Itga2, Itga10*, and *Itgb1* in fibulin-2-deficient 344SQ cells (Figure 6E). Collectively, these findings suggest that tumor cells required fibulin-2 for the creation of a stiff extracellular matrix and adherence to collagen.

## Discussion

Here we posited that fibulin-2, a matrix glycoprotein that functions biomechanically as an inter-molecular clasp, stabilizes tumor extracellular matrix and thereby drives malignant progression. We showed that fibulin-2 was abundant in the extracellular matrix of human lung adenocarcinomas and was distributed in two different intra-tumoral patterns, “fibrillar” and “fibrotic”, suggesting that fibulin-2 may have multiple cellular origins and biological roles. Fibulin-2 was abundantly expressed in metastatic cells from a mouse model of human lung adenocarcinoma and was required for metastatic behaviors of these cells.

Most studies on the role of fibulin-2 in cancer have shown that it functions as a tumor suppressor. The *FBLN2* gene is silenced through methylation or deletion in various tumor types, and reintroduction of fibulin-2 into cancer cells that do not express fibulin-2 reduces motility and invasion *in vitro* [19–22]. The evidence reported here is the first, to our knowledge, to show that fibulin-2 is a driver of malignant progression. Although these experimental findings are unique, they are consistent with reports that fibulin-2 is part of a gene expression signature in primary epithelial tumors that predicts the presence of occult metastasis, and that fibulin-2 may promote the metastatic potential of pancreatic cancer cells through its interactions with MUC4 [10,17]. Thus, fibulin-2 plays a more complex role in cancer than had been previously appreciated.

Fibulin-2 consists of four domains, including the N-terminal domain, three anaphylatoxin modules, a tandem of epidermal growth factor (EGF)-like repeats, and the C-terminal fibulin-type module [8]. In humans and mice, the third EGF-like repeat is either present or absent as a result of alternative splicing in exon 9, and both fibulin-2 isoforms are protein coding [23]. In studies on patients with nasopharyngeal cancer, the spliced fibulin-2 isoform (FBLN2S) is expressed in adjacent normal tissues, is silenced in primary tumor tissues and nasopharyngeal cancer cell lines, is selectively re-expressed in cell lines following 5-azacytidine treatment, and exerts tumor suppressor activity when reintroduced into cells [20]. In KP cells, fibulin-2 western blotting revealed two bands, but analysis of *Fbln2* transcripts revealed only the spliced form, and depletion of fibulin-2 abrogated tumor growth and metastasis, which stands in contrast to tumor suppressor effects of fibulin-2 reported previously [20]. Although the basis for these conflicting findings is unclear, a potential contributor includes cell type-specific biological roles of fibulin-2 in lung and nasopharyngeal cancer cells.

Here we showed that fibulin-2 promotes cross-linking of secreted collagen molecules and tumor cell adherence to collagen, biochemical functions that are presumably unrelated to its structural role as a bridge between matrix molecules [5]. However, the mechanisms by which fibulin-2 performed these biochemical functions are unclear. Fibulin-2 does not associate with collagen type I [24], arguing against a direct role for fibulin-2 in the enzymatic or non-enzymatic steps involved in collagen processing [2]. Unlike tenascin C and periostin, matrix proteins that function as ligands for integrins and other membrane receptors and activate Wnt signaling [25–27], fibulin-2 has no known receptor-binding activity or proven role in the regulation of intracellular signaling. Nevertheless, the prominent effect of fibulin-2 deficiency on gene expression shown here (increased *Loxl2* and decreased collagen-binding integrins and *Loxl4*) implies that fibulin-2 has intracellular roles that have not yet been uncovered.

## Supporting Information

Table S1 **RT-PCR primer sequences.**
(PDF)

Figure S1 **Immunofluorescence staining of fibulin-2 in 344SQ cells stably transfected with control (SCR) or *Fbln2* shRNA #1. Anti-fibulin-2 (red) and DAPI (blue).**
(TIF)

Figure S2 **Co-immunoprecipitation of fibulin-2 and MUC4 in 344SQ cells. After immunoprecipitation with anti-fibulin-2 antibody, western blotting was performed by using anti-fibulin-2 or MUC4 antibodies.**
(TIF)
