# Supplementary material for: Fibulin-2 Is a Driver of Malignant Progression in Lung Adenocarcinoma
Source: PLoS One. 2013 Jun 10;8(6):e67054. doi: 10.1371/journal.pone.0067054 (PMC3677922; doi:10.1371/journal.pone.0067054)
Supplement: Table S1 — RT-PCR primer sequences. (PDF) [file pone.0067054.s001.pdf]

Table S1. RT-PCR primer sequences

gene	forward (5'→3')	reverse (5'→3')
Real-time PCR primers		
Fbln2	CGCAGCTCAACACAGAGCACCG	CTGTGCAATCCTGCCACGGGAC
L32	AACAGGGTGCGGAGAAGGT	TGCTCCCATAACCGATGTTG
Itga1	TGGCTTCTCACCGTTATCCTA	CACACAAGGCATTGATCTCTCT
Itga2	TGTCTGGCGTATAATGTTGGC	CTTGTGGGTTCGTAAGCTGCT
Itga10	CACCAGAGGCCGAATTTGGAT	CCCCAACATGCTGTAAGACAC
Itgb1	CTACTTCTGCACGATGTGATGAT	TTGGCTGGCAACCCTTCTTT
Loxl1	AGGTGCCCGACAACTGGAGAG	CAAGGCCGTCGGAAGGTT
Loxl2	GCCCTCCGATGTGGTCAAG	CCCTCCTTCACCTCCACGTAG
Loxl3	GGAACAGTCTGTGACCGAAAGTG	CACTCAAGTGGATGGCACCCAT
Loxl4	TTCTCGGCTGGCGTTGCTTGTA	GGACTTGGAGAGGCAGTTTTCC
RT-PCR primers		
Fbln2	CAGCCCAACACCTGCAAAG	GACATAGCCTGGCTCACAG
L32	GGACCAAGAAGTTCATCAGG	TCTTAGAGGACACATTGTGAG
