# Supplementary figures and images for: Fibulin-2 Is a Driver of Malignant Progression in Lung Adenocarcinoma
Source: PLoS One. 2013 Jun 10;8(6):e67054. doi: 10.1371/journal.pone.0067054 (PMC3677922; doi:10.1371/journal.pone.0067054)

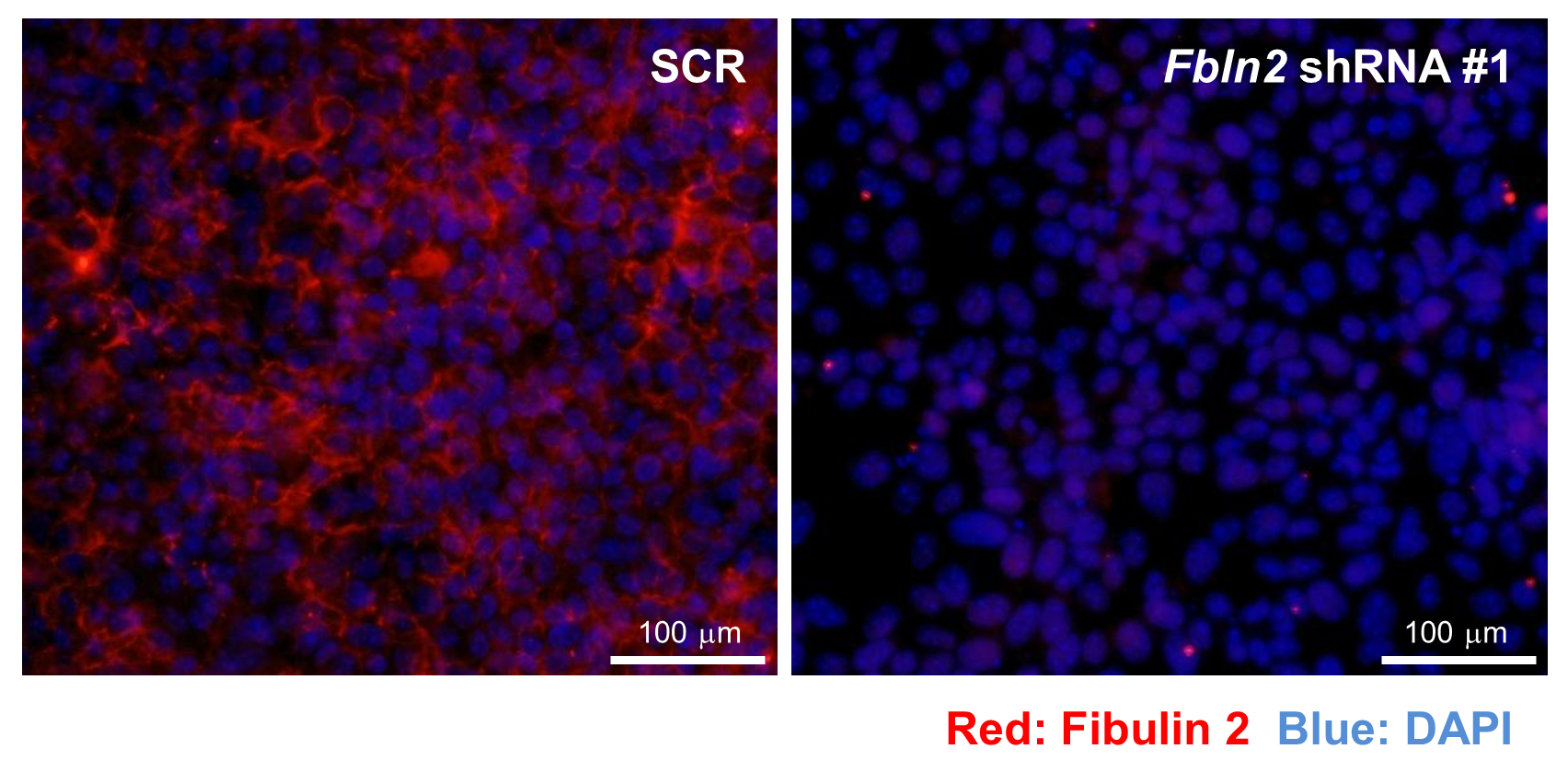

Supplement: Figure S1 — Immunofluorescence staining of fibulin-2 in 344SQ cells stably transfected with control (SCR) or Fbln2 shRNA #1. Anti-fibulin-2 (red) and DAPI (blue). (TIF) [file pone.0067054.s002.tif]

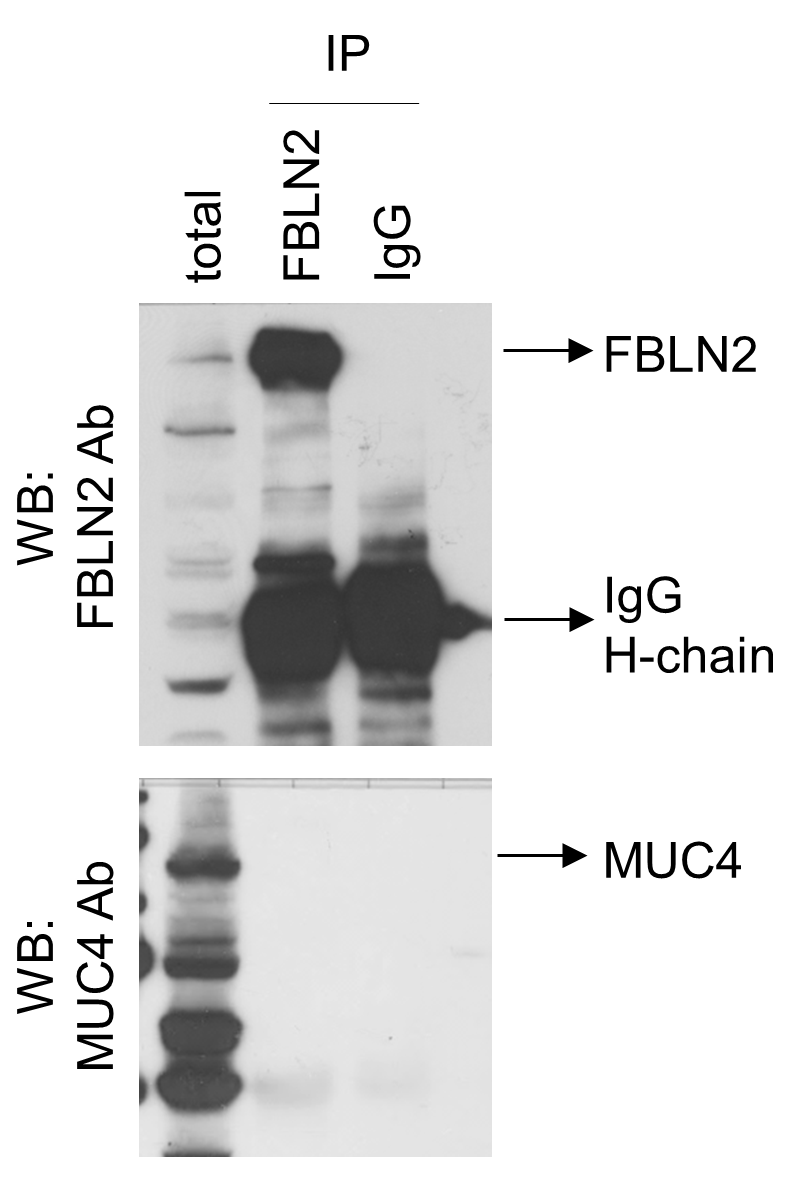

Supplement: Figure S2 — Co-immunoprecipitation of fibulin-2 and MUC4 in 344SQ cells. After immunoprecipitation with anti-fibulin-2 antibody, western blotting was performed by using anti-fibulin-2 or MUC4 antibodies. (TIF) [file pone.0067054.s003.tif]
